# Thermo and Photoresponsive Emulgel Loaded with *Copaifera reticulata* Ducke and Chlorophylls: Rheological, Mechanical, Photodynamic and Drug Delivery Properties in Human Skin

**DOI:** 10.3390/pharmaceutics14122798

**Published:** 2022-12-14

**Authors:** Katieli da Silva Souza Campanholi, Ranulfo Combuca da Silva Junior, Jonas Marcelo Jaski, Jéssica Bassi da Silva, Mariana Carla de Oliveira, Rafaela Said dos Santos, Magali Soares dos Santos Pozza, Lidiane Vizioli de Castro-Hoshino, Mauro Luciano Baesso, Lucio Cardozo-Filho, Marcos Luciano Bruschi, Wilker Caetano

**Affiliations:** 1 Chemistry Department, State University of Maringá, Maringá 87020-900, Brazil; 2 Agronomy Department, State University of Maringá, Maringá 87020-900, Brazil; 3 Laboratory of Research and Development of Drug Delivery Systems, Department of Pharmacy, State University of Maringá, Maringá 87020-900, Brazil; 4 Animal Science Department, State University of Maringá, Maringá 87020-900, Brazil; 5 Physics Department, State University of Maringá, Maringá 87020-900, Brazil; 6 Chemical Engineering Department, State University of Maringá, Maringá 87020-900, Brazil

**Keywords:** emulgel, stimulus-responsive, chlorophylls, cutaneous leishmaniasis, poloxamer, copaiba oil resin, emulsion-filled gel

## Abstract

Recently, the number of new cases of cutaneous leishmaniasis has been of concern among health agencies. Research that offers new therapeutic alternatives is advantageous, especially those that develop innovative drugs. Therefore, this paper presents the incorporation of *Copaifera reticulata* Ducke and chlorophyll extract into Pluronic^®®^ F127 and Carbopol gels, under optimized polymer quantities. The chlorophyll extract (rich in photosensitizing compounds) was obtained by continuous-flow pressurized liquid extraction (PLE), a clean, environmentally friendly method. The system aims to act as as a leishmanicidal, cicatrizant, and antibiotic agent, with reinforcement of the photodynamic therapy (PDT) action. Rheological and mechanical analyses, permeation studies and bioadhesiveness analyses on human skin, and PDT-mediated activation of *Staphylococcus aureus* were performed. The emulgels showed gelation between 13° and 15 °C, besides pseudoplastic and viscoelastic properties. Furthermore, the systems showed transdermal potential, by releasing chlorophylls and *C. reticulata* Ducke into the deep layers of human skin, with good bioadhesive performance. The application of PDT reduced three logarithmic colony-forming units of *S. aureus* bacteria. The results support the potential of the natural drug for future clinical trials in treating wounds and cutaneous leishmania.

## 1. Introduction

In 2020, data provided by the Ministry of Health (DataSUS) revealed 18,000 new leishmaniasis cases in Brazil; of these, 90% corresponded to tegumentary leishmaniasis. Conventional treatments, besides being costly (costs of up to USD 715.35/per treatment with liposomal amphotericin B) [1], require hospital interventions that take 2 to 6 h for drug administration and constant patient monitoring. In this context, the search for efficient, inexpensive, and tolerable therapies has been undertaken by several research groups seeking to treat neglectable diseases [2,3,4,5].

The use of medicinal plants is a means to reduce costs and maintain the effectiveness of treatments. In this respect, the adoption of *Copaifera* genus plants gains prominence in treating leishmaniasis [2,3] by exhibiting activities similar to amphotericin B [6]. Copaiba oil is composed of sesquiterpenes (volatile fraction) and diterpenes (resinous fraction) and is widely employed in skin treatment due to its anti-inflammatory, healing, and antimicrobial properties [7], targeting *Trypanosoma cruzi* [8,9,10] and leishmaniasis [2,3]. The most relevant manifestation refers to the antiprotozoal activity of *Copaífera multijuga*, *Copaífera officinalis*, *Copaífera reticulata* Ducke*, Copaífera lucens*, *Copaífera langsdorfii*, *Copaífera paupera*, *Copaífera martii*, and *Copaífera cearenses* oils against *Leishmania amazonenses* [11]. Biological activity was attributed to the group of sesquiterpenes (especially β-caryoene, α-copaene, zingibereno, β-bisabolene, and bergamothene) and diterpenes (mainly hardwichiic, kovalenic, kaurenoic, polyaltic, and coplic) in different amounts in the species of copaiba oil [11]. Investigations of copaiba oil in scientific publications address formulation methodologies for volatile fraction stabilization, a challenging procedure in drug technology [12].

Topical treatments also require the use of antibiotics to reduce the levels of secondary infection. In this context, antimicrobial photodynamic therapy (PDT) gains prominence because it is a therapeutic modality capable of not developing resistance effects in microorganisms [13,14]. PDT is a widely studied medical modality for the control of antibiotic-resistant strains [15] and fungal and viral inactivations [16,17], and in the regression of brain tumors [18], advanced carcinomas [19], and skin tumors [20] resistant to multidrugs [21,22]. We have previously shown the effectiveness of chlorophyll-rich extracts in PDT, which were capable of causing severe cell wall damage in *Staphylococcus aureus* bacteria [23]. Such extracts were obtained by a clean methodology (green chemistry, with pressurized liquid extraction—PLE), considering sustainability and eliminating waste generation in the environment. Moreover, the extracts showed an optimized composition, with photodynamic activity superior to traditional extraction products (methanol and petroleum ether).

Ideal drugs for dermatological therapy should allow the release of the drugs into the skin, acting as permeation promoters [24]. Emulsion gels allow skin hydration and physical protection, making them useful for the pharmaceutical industry [24]. Pluronic-based stimulus-responsive gels contain hydrophobic microdomains capable of monomerizing chlorophylls and act as promoters of skin permeation [25,26,27], which are mechanisms that are associated with increased hydration and the creation of structural irregularities in the stratum corneum [24,25]. When combining hydrophobic compounds (such as chlorophylls) with emulsions, the preferential migration of nonpolar compounds to oil droplets occurs, allowing the monomerization of photosensitizers and ensuring therapeutic benefits [28].

Given the above, the present study shows the development of a thermal and photoresponsive emulgel composed of copaiba oil microdroplets containing monomerized chlorophylls. The drug aims to contribute to the current therapeutic arsenal and to provide a therapeutic alternative that assists patients with cutaneous leishmaniasis.

## 2. Materials and Methods

Triethanolamine (TEA) was acquired from Synth (São Paulo, SP, Brazil) and Polymer F127 of the Pluronic^®®^ class was acquired from Sigma-Aldrich (St. Louis, MO, USA), while Carbopol C934P^®®^ (Cb) was obtained from Lubrizol Advanced Materials (São Paulo, SP, Brazil). Copaiba oil (*Copaifera reticulata* Ducke—CO) was supplied by the company Copaíba da Amazônia, and was extracted from the conservation area of the Agroextra-Active Association, Aripuana-Guariba (Apuí, AM, Brazil), as previously described [2,3]. Spinach was obtained from the local market (longitude: −51.9375 23°25′38″ S, 51°56′15″ W). Brain Heart Infusion Broth (BHI) and Mueller–Hinton Agar (MHA) were purchased from Himedia (São Paulo, SP, Brazil) and KASVI^®®^ (São José dos Pinhais, PR, Brazil), respectively. All experiments were carried out using ultra-pure water. The use of natural products was registered in SISBIO nº 72922-1 (National Biodiversity Authorization and Information System) and SISGEN nº AE28797 (National Genetic Heritage Management System).

### 2.1. Chlorophyll Extract Obtention

The leaves, stems, and ridges of *Tetragonia tetragonoides* were dried in a circulation oven (model 400/4ND, Ethik Technology, Vargem Grande Paulista, SP, Brazil) at 35 °C, until they had a constant mass (ca 145 h). Then, the dry mass was ground in a knife mill (model SL-30, Solab, Piracicaba, SP, Brazil) and homogenized [29]. Chlorophyll extraction was performed by pressurized liquid extraction (PLE), as described previously [23,29]. In a previous study, we presented a chemometric plan and the mathematical modeling that offered the best conditions regarding the water/ethanol ratio, temperature, and pressure to obtain the highest mass yield. The best-determined extraction condition was 80% (*v*/*v*) of ethanol/purified water, 75 °C, and 80 bar.

#### Extraction Process

**Figure 1:**
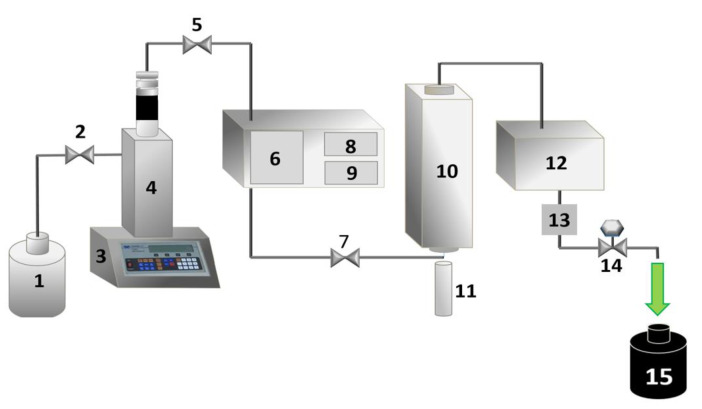
Experimental apparatus for pressurized liquid extraction: (1) solvent reservoir (80% *v*/*v* ethanol/purified water), (2) needle valve, (3) syringe pump controller, (4) syringe pump, (5) needle valve, (6) preheater, (7) needle valve, (8) preheater temperature controller, (9) extractor vessel temperature controller, (10) extractor vessel, (11) extraction cell with 4.0 g of *Tetragonia tetragonoides* (diameter of 1.30 cm and height of 18 cm), (12) cooling system, (13) pressure indicator, (14) “back pressure” valve, and (15) extract collector bottle.

Approximately 4.0 g of *Tetragonia tetragonoides* dry sample was inserted into the extraction cell, which was coupled to an extractor system (Figure 1). The extraction conditions were then adjusted (75 °C and 80 bar), and a fixed period (20 min) was expected. The solvent (80% *v*/*v* ethanol/water) flow rate was 0.75 mL·min^−^^1^, and the exit cooling temperature of the natural extract (Chl) solution was 20 °C. Chlorophyll extract was collected in pre-weighed bottles and protected from light during extraction (4 h). The solvent of the Chl extract was evaporated at 35 °C in a circulation oven, as previously reported [29]. The components of the equipment used in the PLE methodology are shown in Figure 1.

### 2.2. Emulgels Containing C. reticulata Ducke and Chlorophylls

The content of CO incorporated was based on preliminary chemometric planning reported previously. This study found emulgels containing 8, 10, and 12% of *C. reticulata* Ducke in F127 18% *w*/*w* and C934P 0.25% *w*/*w* to be promising for cutaneous leishmaniasis treatment [2]. Therefore, the composition of the intermediate concentration was selected for the addition of chlorophyll extract, generating the emulgels investigated in the present study. The dermatological formulations’ compositions are shown in Table 1 [2,3].

#### Preparation of Emulgels

**Scheme 1:**
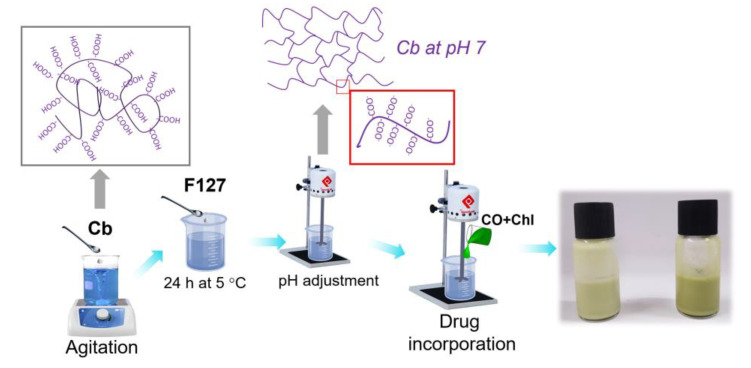
Schematic illustration of EOChl obtained.

Initially, Cb was dispersed in purified water under vigorous stirring (6 h). After this, the solution was added to F127 (Table 1), and the system was maintained at 5 ± 2 °C for 24 h, as shown in Scheme 1. Then, the mixture was stirred (Quimis stirrer, model Q235-2, São Paulo, SP, Brazil) until complete homogenization. Then, the pH was adjusted to 7 using TEA. Next, the CO and chlorophyll extract were mixed, and this oily mixture was slowly added to the gel preparation, which remained in constant agitation for 30 min. Finally, the EOChl-0.5 and EOChl-1.0 (emulsions of copaiba oil and chlorophyll extract) obtained were stored at 5 ± 2 °C for at least 24 h before analysis.

Formulations containing chlorophyll extract and without *C. reticulata* Ducke were obtained for permeation and bioadhesion assays on human skin (ex vivo). A certain amount of oil resin was added to the amount of purified water listed in Table 1 (81.25g of water for EChl-0.5 and 80.75 g for ECh-1.0), and we obtained EChl-0.5 and EChl-1.0. In addition, the standard gel (named Std-gel), without any drug, was also acquired for the permeation studies on human skin.

### 2.3. Mechanical and Rheological Analysis

Mechanical and rheological characterizations were performed at 5.0, 25.0, and 35.0 ± 0.1 °C in order to predict storage-, room-, and skin-temperature behavior.

#### 2.3.1. Rheological Properties

In this study, the measurements were carried out using a commercial rheometer model, HAAKE MARS II (Thermo Fisher Scientific, Karlsruhe, Germany), which contains a parallel steel cone-plate geometry of 35 mm (cone code L09006 C35/2° Ti L, with 0.105 mm of gap separation). The EOChl gels were placed on the analysis support and rested for 1 min (stabilization before analysis). In continuous mode, the upward and downward curves were acquired (shear rate from 0 to 800 s^−1^, with progressive increase in time up to 150 s, kept at the upper limit for 10 s, and then decreasing for 150 s).

In the oscillatory rheometric study, the viscoelasticity of the systems was investigated. The data were obtained in the frequency sweep range from 0.1 to 10.0 Hz in the linear viscoelastic region (L.V.R.). The RheoWin 4.10.0000 (Haake^®®^) software provided the values of the parameters: elastic modulus (G′), viscous modulus (G″), tan δ (determined by G″/G′), and η′ (dynamic viscosity). All analyses were performed with a minimum of three replicates.

#### 2.3.2. Textural Properties

The texture profile analysis (TPA) was conducted using a TA-XTplus Texture Analyzer (Stable Micro Systems, Surrey, England), equipped with a cylindrical probe (10 mm the diameter) moving at 2 mm·s^−1^. Based on the graphs of the responses, the hardness, adhesion, cohesion, elasticity, and compressibility were determined. The hardness was found on the first positive peak of maximum force. Adhesiveness corresponded to the surface area of the first negative. The elasticity and cohesiveness were expressed by the relationship of the second/first (positive) peaks. All the measurements were performed three times for each of the tests. The results are expressed as the mean ± standard deviation.

### 2.4. Temporal Monitoring of the Emulgel

EOChl-0.5 and EOChl-1.0 with 1, 60, and 270 days of preparation were evaluated in the same texturometer, using the hardness, adhesion, cohesion, elasticity, and compressibility responses. These gels were kept at 5 °C (as a two-phase system), protected from light, and rested until the analysis date [2]. The systems were vigorously shaken by hand and kept at room temperature for at least 3 h before analysis. The tests were performed at 25 ± 2 °C, in triplicate.

### 2.5. Stimuli-Responsive Analysis

The gelation temperature (T_sol-gel_) was determined in the same rheometer described. The oscillatory mode was used, with a temperature ramp from 5.0 to 60.0 °C (heating rate of 10.0 °C min^−1^), within the L.V.R., with a frequency of 1.0 Hz and tension (σ) of 13 Pa. The gelling temperature was determined by the second derivative method of the elastic modulus (G′) as a function of temperature [30]. Each analysis was performed in at least three replicate samples.

### 2.6. Performance of the EOChl on Human Skin (Ex Vivo): Permeation and Bioadhesion

These studies were approved by the ethics committee for human research (under protocol n°. 19902019.1.0000.0104), at the State University of Maringá, PR. The protocols followed the Code of Ethics of the World Medical Association (Declaration of Helsinki) [31,32,33,34,35].

#### 2.6.1. Preparation of Human Skin

After surgical removal (from cosmetic surgery), the human abdomen skin samples were refrigerated in sterile thermal boxes and immediately forwarded to the analysis center. The biological tissue was visually evaluated, and the subcutaneous fat and connective tissue were removed using a surgical scalpel. The skin was then carefully washed with saline solution and subjected to skin permeation and bioadhesive analysis.

#### 2.6.2. Bioadhesion on Human Skin

Following a previously reported methodology [2], the bioadhesive force was determined using the TA-XTplus Texture instrument in tension mode. Initially, the human skin was attached to the appropriate cylindrical support, fixed in the probe. Next, the emulgel was inserted in a cylindrical flask and kept at 34 ± 2 °C. During the analysis, a force of 0.1 N was applied for 30 s, allowing skin contact with the formulation. Then, the probe was lifted with a velocity of 1.0 mm/s, and the force–time curve relationship determined the force required to detach the skin from the EOChl or EChl surface [36]. All systems were evaluated using at least three replicate samples.

#### 2.6.3. Permeation Studies by Fourier Transform Infrared Photoacoustic Spectroscopy (FTIR-PAS)

The skin permeation for EOChl and EChl was assessed using human skin pre-treated as described in Section 2.6.1. First, an emulgel sample was homogeneously placed on the surface with 1 cm^2^ epidermis. Then, the formulate/epidermis contact was maintained for 0.5 and 4 h. Afterwards, spectra were obtained using a Fourier transform infrared spectrometer (Vertex 70v, Bruker Optik GmbH, Ettlingen, Germany) equipped with a photoacoustic detection cell (PA 301, Gasera Ltd., Turku, Finland). The analyses were performed using a resolution of 8 cm^−1^, a spectral range between 4000 and 1000 cm^−1^, and an average of 32 scans. First, the spectra of each sample were obtained by reading the epidermal face (after removing excess non-permeated gel with a paper towel, after 0.5 h of drug/skin exposure). Then, the sample was turned over to analyze the opposite side, the dermal face [2,37].

### 2.7. Photodynamic Inactivation of Staphylococcus aureus Bacteria

#### 2.7.1. Microorganism and Culture Conditions

*Staphylococcus aureus* (ATCC 25923) was used in PDT assays. For all tests, the bacteria were previously replicated three consecutive times in test tubes containing 3 mL of BHI broth for 24 h at 37 °C. For the tests, the cell density was standardized in tubes containing 0.9% sterile saline solution, and the turbidity was equivalent to that of the reference tube, using the McFarland scale, which corresponded to 10^8^ colony-forming units (CFU)/mL.

#### 2.7.2. Microbiological Analysis

**Figure 2:**
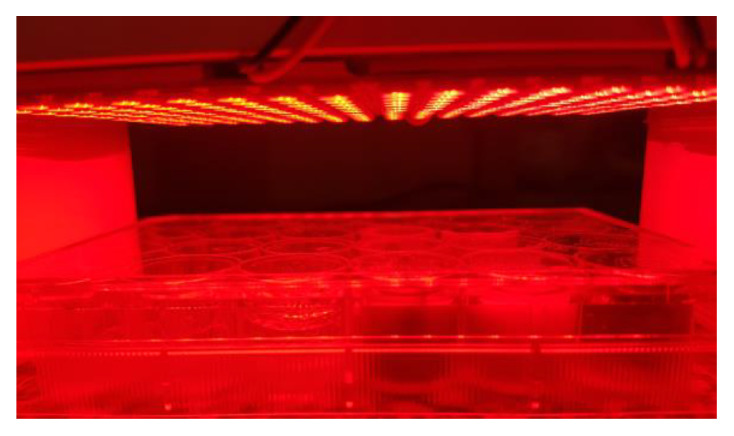
Illumination of EOChl-0.5 gels.

Around 1 mL of Mueller–Hinton broth and 3g of EOCh-0.5 were added in each well of a 12-well plate. Then, the components were mixed using a sterile spatula. After this, 100 μL of the *S. aureus* suspension was added to each plate. Next, the 12-well plates were illuminated for 0.5 h, using a red LED (1.2 mW cm^−2^ with a light dose of 2.15 J cm^−2^, Figure 2). After illumination, 1 mL of the sample was transferred to a petri dish, and seeding was performed by the pour plate method using MH agar. The plates were incubated for 24 h at 37 °C. The controls were performed considering bacteria (without emulgel) and EOChl-0.5 without light. The analysis was performed in triplicate. After incubation, the total bacterial count in CFU/mL was determined [37,38].

### 2.8. Statistical Analysis

The averages were compared using the free software R, version 3.6.0, with the R Studio interface, version 1.1.463 [39]. The statistical test was applied to compare the effect of temperature, copaiba oil, and chlorophyll extract on the thermoresponsive gel properties in terms of the oscillatory rheological behavior (at representative frequencies: 0.316, 1.000, 3.162, and 10.000 Hz), as well as the flow index, consistency index, yield value, hysteresis area, compressibility, adhesion, elasticity, hardness, and cohesiveness parameters. In addition, a statistical analysis was performed to evaluate the results of the photodynamic inactivation of *S. aureus* bacteria. The significance level to reject the null hypothesis was 5% (*p* < 0.05).

## 3. Results

### 3.1. Chlorophyll Extract Features

Details of the extraction and optimization of the experimental conditions have recently been presented [23,29]. The natural extracts obtained from the pressurized liquid extraction (PLE) contained high chlorophyll *a* and *b* levels and were, therefore, more effective in PDT [23]. Moreover, the PLE methodology yield was approximately twice the conventional one (15% *w*/*w* for the conventional method [40] and 25% for PLE extraction optimized [29]). In addition, PLE extractions do not generate waste in the environment and are fast (extraction plateau between 60 and 100 min), while traditional extractions generate large amounts of toxic waste and require several days of operation, as previously reported [29]. Additionally, we showed the antibiotic potential of the chlorophyll extract obtained from PLE and a conventional methodology [23], data that further verify the product used in the present work.

### 3.2. Macroscopic Features

**Figure 3:**
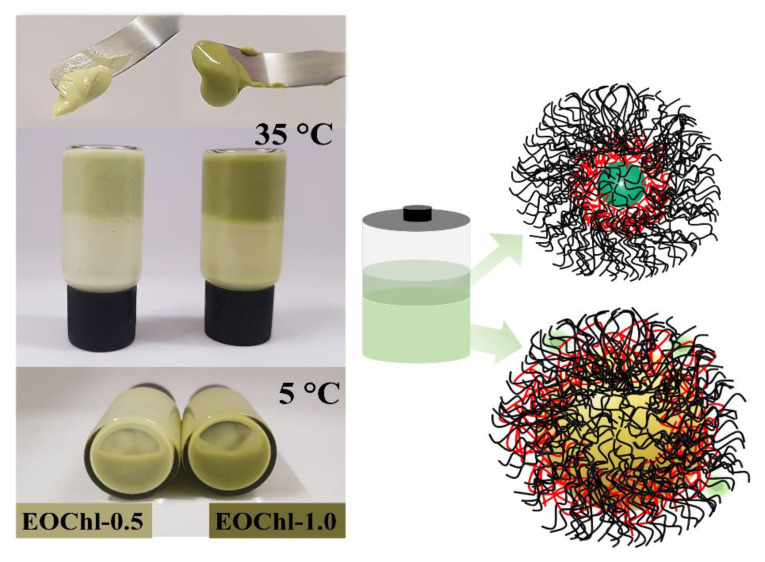
Image of emulgel system at 5 °C and 35 °C and the phase separation representation with micellar dynamics.

The formulations were opaque, with the absence of phase separation when stored at room temperature (Figure 3). However, storage at low temperatures allowed the mobility of polymeric micelles due to the thermoresponsive property, leading to the formation of a biphasic system after 15 days [41]. In the creaming sedimentation, one surface phase was translucent, with an intense green color; meanwhile, the bottom phase was opaque and had mild green coloration. This behavior can be justified by the migration of chlorophylls (and derivatives) to the hydrophobic portions of the micelles, which competes with oil stabilization. Thus, two main populations of micellar systems are formed: one contains oil droplets and chlorophyll on the microscopic scale, and the other is translucent, containing chlorophylls and a small amount of oil on the nanometric scale (colorless region). Nevertheless, phase separation did not impair the performance of the gel, and the creaming sedimentation had reversible behavior. Moreover, when stirred, the emulsion was able to return to its homogeneous condition. This behavior could be verified by the mechanical properties of the emulgels after prolonged storage, as discussed in later sections.

### 3.3. Stimuli-Responsive Properties

**Figure 4:**
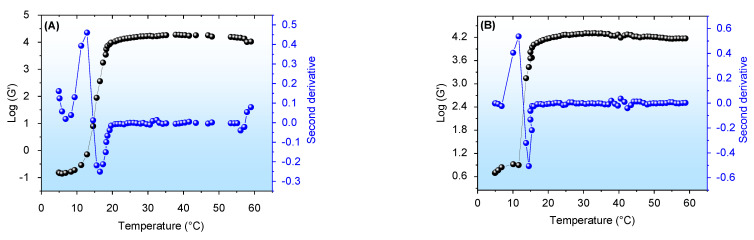
Logarithm of elastic modulus (G′ in Pa) as a function of temperature, with the corresponding second derivative being (**A**) EOCh-0.5 and (**B**) EOCh-1.0 emulgel. Standard deviations were omitted for clarity; however, in all cases, the coefficient of variation of the replicate analyses was <10%.

The thermo-stimulated properties aims to allow the easier administration of the dermatological platform (since it is in a liquid state at low temperatures) and longer permanence on the wound surface, after thermal body stimululation. We have previously presented an emulgel composed of F127 18%, C934P 0.25%, and *C. reticulata* Ducke 10% *w*/*w* [2] with a T_app-gel_ value (apparent gelling temperature) near 12 °C. Similarly, gels composed of chlorophyll extract in F127/C934P gel had a gelation temperature close to 27 °C [40]. The stimuli-responsive behavior of the EOCh emulgels is shown in Figure 4.

Figure 4 shows the curves obtained by the logarithm of elastic modulus (G′) by temperature increase. In these analyses, the viscosity of the formulations also followed a sigmoid profile analogous to the G′ behavior. EOCh-0.5 and EOCh-1 demonstrated low viscosity at low temperatures (Figure 4A,B), due to the formation of hydrogen bonds that solvated the micellar chains. However, with temperature increases, the F127 hydrophilic chains (polyethylene oxide (PEO)) became dehydrated, and the emulsion was restructured at the molecular level. A hydrophobic interaction (among polypropylene oxide (PPO) chains) is favored in this process, which causes an increase in viscosity due to the friction between the polymer chains [42]. For these emulsions, the oil interface/polymer interactions also increased the overall viscosity of the formulations, as we previously showed [2,43].

After the complete micellar rearrangement thermodynamics, the gel viscosity remained constant and independent of the temperature. However, slight variations could occur due to compositional changes related to the loss of volatile components in the resin oil [30,44]. In the F127/C934 systems, typically, in the vicinity of the T_sol-gel_ temperature, a change from elastoviscous (G′ < G″, until the T_sol-gel_) to viscoelastic (G′ > G″, after the T_sol-gel_) occurs [45,46], i.e., G′ intersects G″ as a characteristic of the gelation process of these systems. However, as displayed in the previous study [3], CO emulgel does not show an interception of its curves. Thus, the sigmoidal behavior is related to the changes that occur due to micellar thermodynamics, and the emulgels showed predominant viscoelastic behavior over all temperature scanning. The T_sol-gel_ values obtained were statistically similar (*p* > 0.05), being 15.0 ± 1.4 °C for EOCh-0.5 and 13.3 ± 0.6 °C and EOChl-1.0. Of these results, all the EOCh samples exhibited a thermo-stimulated viscosity increase, which demonstrated their ability to form a consistent and protective layer on the open wound after administration, when applied at low temperatures (after removal from refrigeration and promptly applied to skin).

### 3.4. Evaluation of Flow Properties of Emulgels

**Figure 5:**
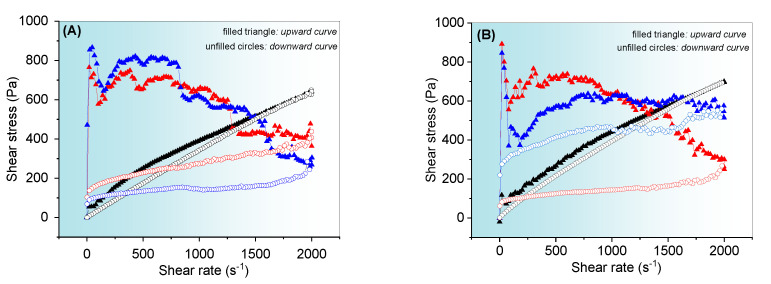
The flow curves of (**A**) EOCh-0.5 and (**B**) EOCh-1.0 systems at different temperatures. The symbols in blue correspond to measurements at 37.0 °C, red at 25.0 °C, and black at 5.0 °C. The filled triangles correspond to the upward curve, and the open circles (with borders of the same color) to the respective downward curve. Standard deviations were omitted for clarity; however, in all cases, the coefficient of variation of the replicate analyses was <10%.

The evaluation of rheological properties by continuous flow shear provides information on technical aspects, such as viscosity variation during industrial production and the transportation of the formulation to the storage facility or sales center. Moreover, the evaluation of the hysteresis area shows viscosity changes during the shear stress caused by friction during administration, which can favor drug release. The flow behavior of the formulations is shown in Figure 5.

The flow curves showed a significant variation before (5.0 °C) and after the gelling temperature (25.0° and 37.0 °C) was applied. Moreover, the curves in Figure 5A,B show behavior indepdent of the chlorophyll extract’s concentration. The EOChls showed pseudoplastic behavior [40,47,48], since the relationship between stress and the rate was not linear (non-Newtonian, n < 1). This result is due to the higher initial viscosity of the gel as a result of the molecular randomization state of the polymer chains in rest, which results in a steep slope in the flow curves under low shear stress conditions. Nevertheless, the polymeric chains become aligned at a molecular level with the shear stress effect, and the profiles gradually become less steep. Moreover, the emulsion droplets align themselves in the direction of shear and become smaller with friction [2]. Droplet size redistribution during shear was favored at 25.0 and 37.0 °C due to the high viscosity, and it did not allow modeling by the power law and Herschel–Bulkley [2,3].

Additionally, the emulgels at temperatures of 25.0 and 37.0 °C showed a highly positive hysteresis area (absence of overlap of each upward and downward curve) [49], which characterized the system as thixotropic. The thixotropy indicates the inability of the platform to recover its original state within the time interval of the analysis, a situation concordant with the changes in the droplets. Thixotropic emulgels release drugs in a rapid manner due to their low viscosity after shear [50].

At 5.0 °C, complex flow behavior was not observed, probably because of the low viscosity of the system. The small hysteresis area, under this condition, suggests the uncoiling effect of the polymeric network at low temperatures, and it requires a longer relaxation time to obtain the initial random structure. This result was in agreement with the previously obtained result for the copaiba gel at 5.0, 25.0, and 37.0 °C [2,3] and other reported emulgels [51].

### 3.5. Evaluation of Viscoelastic Properties of Emulgels

**Figure 6:**
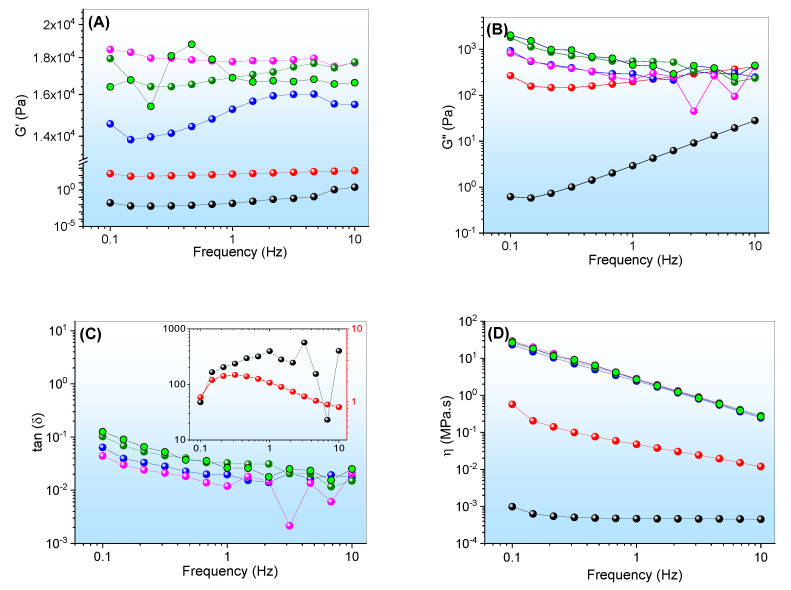
Viscoelastic properties of formulations in terms of oscillatory frequency: (**A**) elastic moduli (G′); (**B**) viscous moduli (G″); (**C**) loss tangent (Tan δ); (**D**) dynamic viscosity (η′). The symbols refer to ● EOCh-0.5 at 5.0 °C, ● EOCh-1.0 at 5.0 °C, ● EOCh-0.5 at 25.0 °C, ● EOCh-1.0 at 25.0 °C, ● EOCh-0.5 at 37.0 °C, and 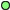 EOCh-1.0 at 37.0°C. The insert (**C**) corresponds to the viscoelastic data at 5.0 °C.

The preparations were also evaluated by small-amplitude oscillatory rheology (within the linear viscoelastic region) as a function of three temperatures (Figure 6). Under this condition, the infinitesimal deformations kept the polymeric chains close to equilibrium, and the responses provided molecular-level interaction information.

The formulations showed an elastic modulus (G′) higher than the viscous modulus (G″) throughout the frequency sweep at 25.0° and 37.0 °C, which provided tangent loss values (tan δ) of less than one unit (Figure 6A–C). The EOCh systems presented viscoelastic properties, which indicated a highly structured system, with great interactions among their constituents. Viscoelastic behavior is usually recommended for pharmaceutical products, in order to improve their retention at the site of application [40,52,53]. G” exceeded G′ in both gels at 5.0 °C, therefore indicating elastoviscous systems with a predominantly plastic nature. Elastoviscous to viscoelastic conversion has already been reported for gels containing *C. reticulata* Ducke [2].

Additionally, the G′ and G″ values revealed a low dependence on frequency, which indicated a rigid structure that did not undergo structural rearrangements during the test [54]. However, the preparation containing 1% (*w*/*w*) of chlorophyll extract, at 37.0 °C, displayed G′ variation below 1 Hz, which suggests a system weaker than the others. This behavior is in agreement with the low gelation temperature observed for this system, which confirms the significant structural changes with chlorophyll saturation.

Emulgels composed of other oils, such as andiroba oil, have already been demonstrated for G′ > G″, over a range of frequencies [43]. In the same way, copaiba oil resulted in emulsion preparations with predominantly viscoelastic behavior after the gelation process. Furthermore, the dynamic viscosity (η′) of the systems decreased as the frequencies increased. At 5.0 °C, they displayed lower viscosity values compared to 25.0 and 37.0 °C, which is in agreement with the gelation temperatures of these systems. Above the micellization of the F127, the polymers were highly organized, thus leading to elevated G′ values and improving the interactions among the constituents of the formulation.

### 3.6. Evaluation of Mechanical Properties

The TPA method can be used to characterize semi-solid formulations and correlate them with clinical efficacy [55]. Through the force–distance graphical analysis, the parameters of hardness, compressibility, adhesiveness, cohesiveness, and elasticity were obtained [56]. The data are shown in Table 2.

Hardness and compressibility refer to the force and work required for the deformation of the EOChl. The knowledge of these parameters helps to clarify the behavior of the EOChl during the administration step [55,56]. Low hardness and compressibility aid in the easy removal of the drug from the storage bottle and facilitate its administration. EOChl-0.5 and EOChl-1.0 displayed a significant increase (*p* < 0.05) in hardness and compressibility with the elevation of the temperature from 5° to 25 °C. This behavior occurs due to the change in micellar structure with the thermodynamics of the F127. On the other hand, the temperature increase from 25° to 37 °C did not exert a significant influence (*p* > 0.05) on these parameters. Additionally, when comparing EOChl-0.5 and EOChl-1.0 at 37 °C, we noted a significant difference (*p* < 0.05) in hardness and compressibility, in which EOCh-0.5 exhibited higher values of these parameters than EOChl-1.0. This result shows that the chlorophyll extract at higher concentrations makes the interactions among the polymer chains weaker, which is in agreement with the rheological results.

The adhesiveness was evaluated by the attractive force between the sample and the probe surface used in the analysis [55]. The adhesiveness increased with temperature elevations for both emulgels (*p* < 0.05). However, at 5 °C, none of the formulation present adhesion values.

Regarding the comparison of EOChl-0.5 and EOChl-1.0 at 25 °C, there was no significant difference (*p* > 0.05) in adhesiveness, while, at 37 °C, there was a significant difference (*p* > 0.05) between them. An exact correlation was observed in the values of hardness and compressibility, as discussed previously; thus, these parameters (hardness, compressibility, and adhesiveness) can be correlated and explained by the increase in the viscosity of the samples with temperature [56].

The elasticity of emulgels reflects their ability to reorganize their structures, while cohesion reflects the magnitude of the interactions among the polymer chains. The elasticity and cohesiveness results showed no significant difference (*p* > 0.05) among EOChl samples and temperatures (5, 25, and 37 °C). Moreover, the elasticity values showed that the emulgels underwent structural reorganization quickly. Furthermore, the high cohesiveness values demonstrated adequate attractive forces in the emulgels, analogous to other reported studies [2,37,57].

### 3.7. Temporal Monitoring of the Emulgel

Although the gels exhibited phase separation when stored at low temperatures, the stability studies showed that the mechanical benefits were maintained after storage and manual stirring. The data in Table 3 refer to the emulgels at 1, 60, and 270 days of preparation time.

The textural properties (Table 3) were maintained at values statistically equivalent to the first day of preparation (*p* > 0.05). The lower precision verified on day 270 was linked to the airtight conditions of the storage bottle, which led to the loss of a small amount of water from the pharmaceutical composition. Therefore, in this case, the two-phase behavior did not reduce or impair the performance of the pharmaceutical product.

### 3.8. Bioadhesion and Permeation in Human Skin

Bioadhesion results from the gels’ ability to interact with biological tissues. Topical bioadhesive and viscoelastic systems are of great interest in treating lesions, as they allow the drug to be exposed for a prolonged time on the wound [58]. Furthermore, the adhesiveness reflects the tendency of the gel to form a protective and healing film on the injury surface, allowing administration for longer time intervals [59,60], which minimizes the cost of treatment.

The properties provided in the assays comprise the bioadhesion force (BF—maximum detachment force between the analytical probe and the skin) and the bioadhesion work (BW—detachment energy involved), as shown in Table 4.

Copaiba oil caused a 17% reduction in BF, when compared to EChl and EOChl, which contained 0.5% chlorophyll (*p* < 0.05). On the other hand, the oil resin incorporation did not result in significant changes in the systems containing 1.0% chlorophyll extract (*p* > 0.05). The BW was statistically similar for all evaluated compositions (*p* > 0.05). We previously reported bioadhesive values on human skin of 0.06 to 0.08 N for formulations containing 8 and 12% *w*/*w* of copaiba oil [2]. Therefore, adding chlorophyll increases the overall bioadhesiveness, which optimizes the performance of EOChl systems. This behavior may be linked to the higher apolar content in the formulations, which favors the interaction with skin for drug partitioning effects. Furthermore, the F127/Carbopol polymer blend can establish different physical, chemical, and mechanical interactions (entombment effects and/or polymer interpenetration) with human skin, which allows the intrinsic adhesiveness of these systems [61]. Thus, when administered to damaged skin with moist surfaces and irregular topology, it promotes more outstanding adhesion due to the hydrophilic nature of the PEO micellar segments and Carbopol^®®^ carboxylic groups, which allow interdiffusion effects of the gel chains and the biological segments [58]. Similar bioadhesion results have been reported for emulgels composed of andiroba oil [62].

**Figure 7:**
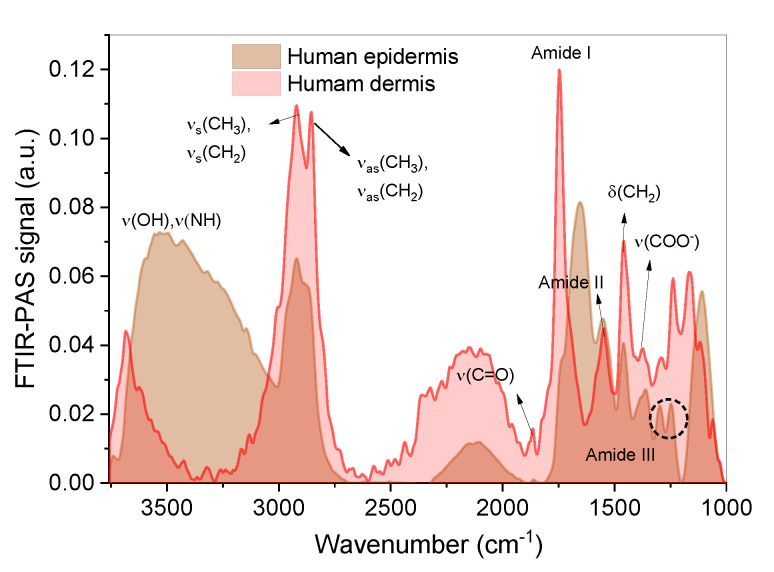
FTIR-PAS absorption spectra for human skin. Note: vibrations—ν: stretching (symmetric or asymmetric) and δ: angular deformation.

After establishing the bioadhesive potential on human skin, permeation studies were conducted via FTIR-PAS spectra. This study provides information about the ability of drugs to overcome the stratum corneum barrier and reach the deep layers of the skin [63,64,65]. We showed previously that chlorophyll extract reaches the dermis after 0.5 h of epidermal administration [40]. We also reported the permeation effects of *C. reticulata* Ducke on human skin, which depends on the droplet size of the emulgel [2]. Moreover, it was found that F127 acts as a permeation promoter, by increasing the permeation rate of *C. reticulata* Ducke [2]. Figure 7 shows the FTIR-PAS spectra for the human epidermis and dermis.

**Figure 8:**
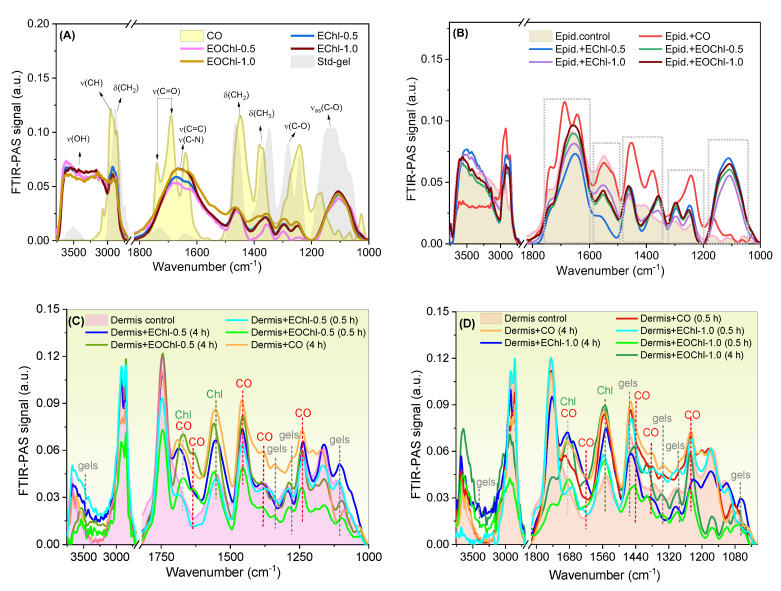
Absorption spectra obtained by FTIR-PAS: (**A**) CO and emulgels; (**B**) human epidermis after 0.5 h of emulgel administration; (**C**) human dermis after 0.5 h and 4 h of administration of the emulgels and CO; (**D**) human dermis after 0.5 h and 4 h administration of EChl-1.0, EOChl-1.0, and CO. Note: vibrations—ν: stretching and δ: angular deformation.

Figure 7 shows the typical spectra of the epidermis of human skin, with signs of lipid (from 3517 cm^−1^ to 2700 cm^−1^), protein, and collagen components (from 1870 cm^−1^ to 1000 cm^−1^) [66]. The human epidermis and dermis spectra were similar, with additional low-intensity signs in the human dermis related to lipid and protein groups [2,66]. The FTIR-PAS spectra of the emulgels and CO are shown in Figure 8A. After medicinal administration on the skin, the reading of the epidermis was performed after 0.5 h. The reading on the opposite side of application (human dermis) was performed after 0.5 h and 4 h (Figure 8C,D).

The emulgels displayed signals around 1130 cm^−1^ (ν_as_C-O) and 3626 cm^−1^ (νO-H), which are related to the F127 and Carbomer chains (Figure 8A). The CO spectra showed the typical natural vibration frequencies of sesquiterpenes and diterpenes, as revealed previously [67,68,69,70,71]. The main signs of CO were preserved after the formulation process (1800–1600 cm^−1^), with displacement and enlargement effects. These data are in agreement with our reported studies [2] and they suggest the preservation of the volatile and fixed components after gel obtention [12], as we have shown previously in validation studies of the same dermatological platform with *C. reticulata* Ducke, albeit without chlorophyll addition [2]. The most prominent chlorophyll peaks were verified from 3000 cm^−1^ to 2902 cm^−1^, which indicate CH_3_ and CH_2_ vibration, and 1650 cm^−1^ for C-N vibration for porphyrins [72].

Typical signs of the emulgels were displayed on the epidermis (Figure 8B), as highlighted in the spectra, which showed permeation in this superficial tissue layer. The signals were intense for CO, due to its more significant interaction with the stratum corneum’s apolar segments.

Dermis analysis (Figure 8C,D) showed the permeation of all formulations and CO, suggesting that this is a transdermal medicine. When increasing the exposure time of the systems on the skin, the signs of permeation increased as well, thus showing, after 4 h, more intense peaks typical of polymers, chlorophylls, and CO. Although the CO (pure) has shown remarkable partitioning effects, the formulation increases the exposure time of the drug on the wound and exerts physical protection and antibiotic effects. Furthermore, it is noteworthy that the permeation intensities of the EOChl-0.5 and EOChl-1.0 after 4 h were similar (Figure 8C,D), thus suggesting an immediate and equivalent therapeutic effect. The permeation profiles’ similarity is in line with the bioadhesion, which has also been shown to be similar for the EOChl emulgels. The variations in peak intensity in the 1000 and 1200 cm^−1^ regions in the dermis spectra collected after 0.5 and 4 h may be linked to the structural changes in the skin or drug saturation over time.

### 3.9. Antimicrobial Photodynamic Inactivation

In previous studies, we developed a bioadhesive gel containing 8 to 20% *C. reticulata* Ducke [2,3,57]. In general, the emulgel showed antimicrobial properties, satisfactory healing capacity verified in vivo studies, fly repellency (preventing myiasis) [57], and antiprotozoal capacity [2]. We also demonstrated the antimicrobial potential of the chlorophyll extract obtained by the PLE methodology, which caused severe damage to the cell wall of *S. aureus* bacteria with low concentrations of the extract (2–4 mg·mL^−1^) [23]. The combination of CO with chlorophyll extract aims to increase the spectrum of activity, favoring healing [57] and microbicidal treatment [23], with the benefits of not generating the microbial resistance typical of PDT [73]. The use of the extract in PDT has already been validated in our previous study, which showed equivalent singlet oxygen quantum yields for pure chlorophyll *a* (Φ_∆_^1^O_2_ = 0.51) and chlorophyll extract (from spinach) (Φ_∆_^1^O_2_ = 0.58) in ethanol [40].

**Figure 9:**
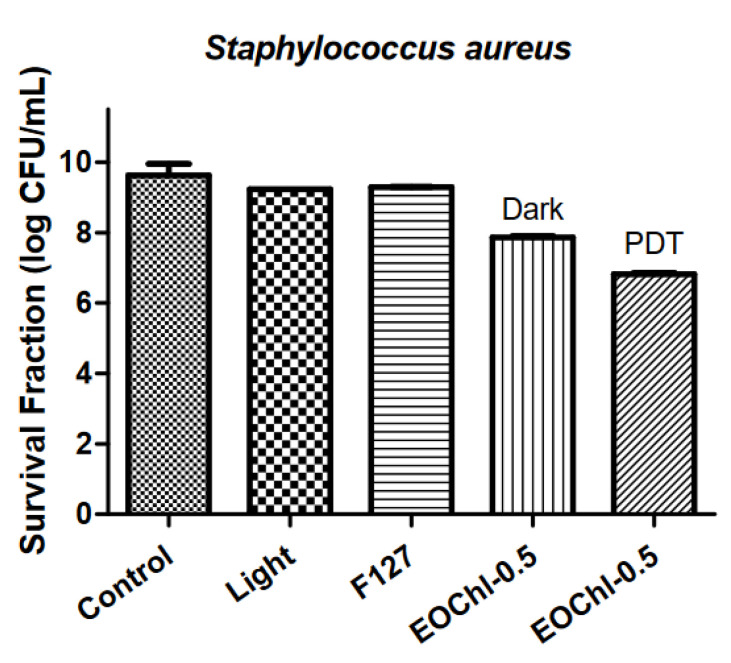
Total *S. aureus* count expressed in log CFU/mL.

The antimicrobial activity of EOCh-0.5 was determined to verify the effect of the drug combination (Figure 9).

The red light and F127 polymer showed equivalent counts in terms of bacteria control (*p* > 0.05). Moreover, there was a significant difference between the control (log 9.63 CFU/mL) and EOChl-0.5 without light (log 7.86 CFU/mL) (*p* < 0.05), which was mainly related to the bacterial potential of CO [57] and chlorophyll in the dark. Some authors have reported the bactericidal and bacteriostatic activity of chlorophyll; however, its mechanism of action on microorganisms in the absence of light is unknown [74]. Possibly, this activity is linked to the positively charged peptidoglycan barrier of *S. aureus* bacteria, which facilitates its complexation with chlorophylls, which have a negative charge density at pH 7.

With red light on EOChl-0.5, the photosensitizers were activated and their effect against *S. aureus* increased. The EOChl-0.5 PDT showed log 6.82 CFU/mL, meaning a reduction of 3 logarithmic units (Figure 9). Comparison of EOChl-0.5 with and without PDT showed a statistically significant variation (*p* < 0.0001). This difference demonstrates the relevance of photodynamic mechanisms, in which radical oxygen species are formed (especially singlet oxygen); these are responsible for killing bacteria without developing bacterial resistance effects. Therefore, 10% CO combined with 0.5% *w*/*w* of chlorophyll extract increased the system’s bactericidal potential, making it more promising for dermatological applications.

## 4. Conclusions

The emulgels exhibited thermoresponsive properties, with gelation between 13 and 15 °C. The systems were pseudoplastic and viscoelastic at body temperature. Hardness, compressibility, and adhesiveness increased with temperature elevation, which may favor the longer permanence of the gel at the treatment site. Phase separation at low temperatures was reversible and did not impair the performance of the systems. The emulgels were able to release the chlorophylls and copaiba oil into the deep layers of human skin, with bioadhesion values applicable to topical delivery systems. The application of photodynamic therapy allowed a reduction of three logarithmic units in the colony-forming units of *Staphylococcus aureus* bacteria. This set of results shows the potential of the developed formulation and makes it a promising candidate for future clinical trials in treating wounds and cutaneous leishmaniasis.

## Figures and Tables

**Table 1:** Dermatological gel composition for 100 g.

Composition	Formulation	
	EOChl-0.5 (g)	EOChl-1.0 (g)
F127	18.0	18.0
Copaiba oil (CO)	10.0	10.0
Carbopol	0.25	0.25
Chlorophyll extract (Chl)	0.5	1.0
Purified water	71.25	70.75

**Table 2:** Mechanical properties—hardness (N), compressibility (N·mm), adhesiveness (N·mm), elasticity (dimensionless), and cohesiveness (dimensionless)—of formulations at different temperatures.

Parameters	EOChl-0.5			EOChl-1.0		
	5 °C	25 °C	37 °C	5 °C	25 °C	37 °C
Hardness	0.07 ± 0.01	1.36 ± 0.07	1.41 ± 0.07	0.08 ± 0.02	1.37 ± 0.04	1.27 ± 0.02
Compressibility	0.49 ± 0.07	13.68 ± 0.82	14.28 ± 0.60	0.57 ± 0.10	13.97 ± 0.58	12.67 ± 0.09
Adhesiveness	-	11.79 ± 0.66	12.14 ± 0.38	-	12.21 ± 0.49	10.99 ± 0.06
Cohesiveness	0.93 ± 0.06	0.96 ± 0.03	0.94 ± 0.01	0.96 ± 0.01	0.96 ± 0.01	0.94 ± 0.01
Elasticity	0.98 ± 0.04	0.99 ± 0.00	1.00 ± 0.00	1.00 ± 0.00	1.00 ± 0.00	1.00 ± 0.00

**Table 3:** Mechanical properties at 25 °C, obtained 60 and 270 days after preparation.

	EOChl-0.5			EOChl-1.0		
	Days					
Parameters	1°	60°	270°	1°	60°	270°
Hardness (N)	1.34 ± 0.06	1.46 ± 0.05	1.35 ± 0.70	1.35 ± 0.05	1.64 ± 0.07	1.45 ± 0.39
Compressibility (N·mm)	6.84 ± 0.42	6.69 ± 0.67	6.61 ± 3.41	6.92 ± 0.37	7.32 ± 0.61	6.77 ± 1.67
Adhesiveness (N·mm)	6.02 ± 0.32	5.83 ± 0.43	5.77 ± 2.56	6.12 ± 0.29	6.29 ± 0.47	5.80 ± 1.06
Elasticity (dimensionless)	0.99 ± 0.02	1.01 ± 0.01	1.00 ± 0.00	0.99 ± 0.00	1.03 ± 0.04	0.99 ± 0.02
Cohesiveness (dimensionless)	0.95 ± 0.04	0.94 ± 0.03	0.97 ± 0.10	0.96 ± 0.01	0.92 ± 0.02	0.94 ± 0.04

**Table 4:** Bioadhesion in human skin at 35.0 °C.

Bioadhesion Parameter	Formulation			
	EChl-0.5	EChl-1.0	EOChl-0.5	EOChl-1.0
BF—Force (N)	0.090 ± 0.005	0.092 ± 0.003	0.075 ± 0.004	0.094 ± 0.010
BW—Work (N·sec.)	0.011 ± 0.003	0.011 ± 0.001	0.011 ± 0.003	0.013 ± 0.001

## Data Availability

Not applicable.
